# Perioperative blood loss is a risk factor for postoperative delirium in geriatric hip fracture patients: a retrospective study

**DOI:** 10.3389/fmed.2025.1617891

**Published:** 2025-07-25

**Authors:** Yubo Deng, Tianqin Zhang, Hu Xie, Jingshan Zeng

**Affiliations:** The Second People’s Hospital of China Three Gorges University, Yichang, China

**Keywords:** postoperative delirium, hip fracture, geriatrics, blood loss, risk factors

## Abstract

**Background:**

Postoperative delirium (POD) is a common and serious complication in elderly patients with hip fractures and is associated with adverse outcomes. The aim of this study was to investigate perioperative blood loss as a potential risk factor for POD.

**Methods:**

This retrospective cohort study included electronic medical records of hip fracture patients admitted to Yichang Second People’s Hospital from January 2020 to June 2024, with a total of 247 patients. POD was diagnosed using the Confusion Assessment Method (CAM) on the basis of the Diagnostic and Statistical Manual of Mental Disorders, Fifth Edition (DSM-5) criteria. Perioperative blood loss was calculated using the Gross linear equation for red blood cell volume, and preoperative blood volume (PBV) was estimated using the Nadler formula. Univariate and multivariate logistic regression analyses were performed to identify independent risk factors for POD.

**Results:**

The incidence of POD was 39.7% (98/247). Multivariate analysis revealed that increased intraoperative blood loss (OR: 6.017, 95% CI: 3.325–10.887, *p* < 0.001), prolonged surgical duration (OR: 1.072, 95% CI: 1.045–1.101, *p* < 0.001), history of coronary heart disease (OR: 3.175, 95% CI: 1.546–6.519, *p* = 0.002), and history of cerebral infarction (OR: 3.170, 95% CI: 1.546–6.497, *p* = 0.002) were independent risk factors for POD. Binary logistic regression revealed a significant dose–response relationship between blood loss and POD susceptibility (all *p* < 0.05). No significant associations were found with age, sex, or BMI.

**Conclusion:**

This study confirms that perioperative blood loss is an independent and modifiable risk factor for POD in elderly hip fracture patients. On the basis of these findings, optimizing perioperative management—such as reducing intraoperative blood loss and shortening surgical duration—may serve as an effective strategy to lower the incidence of POD in this population.

## 1 Introduction

Postoperative delirium (POD) is an acute, transient neurocognitive disorder that predominantly affects elderly and frail patients, typically manifesting within 1–5 days following surgery, with peak incidence observed within the first 24–48 h after surgery (1). This condition results from transient neuronal dysfunction induced by systemic disturbances, including metabolic imbalances, inflammation, and neurochemical dysregulation (2). Clinically, POD is characterized by disturbances in attention, fluctuating levels of consciousness, and disorganized thought processes (3). POD is particularly prevalent in elderly populations, with the literature reporting its occurrence in up to 50% of hospitalized patients aged 65 years and older (4). Elderly and frail surgical patients often experience more severe and prolonged symptoms, which significantly increase the risk of subsequent long-term cognitive decline, functional impairment, and dementia (5).

POD is associated with a spectrum of adverse outcomes, including prolonged hospitalization, increased health care costs, impaired rehabilitation, diminished functional and cognitive recovery, increased risk of incident dementia, and elevated short- and long-term mortality rates (6–11). A meta-analysis of 71 studies revealed that older inpatients with delirium had a threefold higher mortality risk than did those without delirium (12). Given the aging population and the increasing number of elderly patients undergoing surgery, identifying modifiable risk factors for POD is critical for improving perioperative care.

Previous studies have identified several risk factors for POD in elderly hip fracture patients, including advanced age, preoperative dementia, hypoalbuminemia, diabetes, and surgical delay (13–16). However, these patient-related factors predominantly stem from patients’ baseline conditions or health care system limitations, making them difficult to modify clinically and hindering attempts to alter the occurrence of delirium.

While numerous studies have investigated perioperative blood loss, most have treated it as an outcome measure rather than a contributing factor. There remains a notable research gap examining perioperative blood loss as an independent risk factor. The aim of this study was to further investigate the risk factors and underlying mechanisms of POD in elderly hip fracture patients, with a focus on perioperative blood loss and its association with delirium. By addressing gaps in the current literature, this research seeks to provide a scientific foundation for more effective prevention and management strategies for POD, ultimately improving patient outcomes and reducing the health care burden.

## 2 Materials and methods

### 2.1 Study design and patients

This retrospective cohort study utilized electronic medical record (EMR) data from the Second People’s Hospital of Yichang, China. Between January 2020 and June 2024, a total of 357 patients with hip fractures were admitted. The study protocol was approved by the Medical Ethics Committee of the Second People’s Hospital of Yichang (Approval No. 202523).

Data were collected by researchers through review of patient records obtained from the hospital’s EMR system. The inclusion criteria were as follows: 1. age ≥ 65 years, clinical diagnosis of hip fracture, 3. injury mechanism attributed to low-energy trauma (e.g., falls from standing height), and 4. undergoing surgical treatment. The exclusion criteria were as follows: 1. open fractures or pathological fractures, 2. preoperatively impaired consciousness or delirium, 3. incomplete clinical records, or 4. perioperative mortality.

**FIGURE 1:**
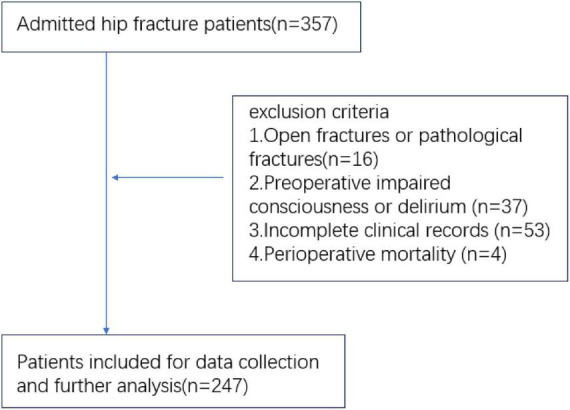
Flowchart of patient enrollment.

After applying the predefined inclusion and exclusion criteria, 247 eligible patients were identified, and their data were extracted for further analysis (Figure 1).

### 2.2 Data collection

Demographic and clinical information was extracted from medical records by the first three authors of this study, with independent cross-verification of all collected data. Any discrepancies or disagreements were resolved by the corresponding author.

The variables were categorized as follows: 1. demographic data: age, sex, and body mass index (BMI); 2. comorbidities (excluding diseases with a prevalence < 10% in eligible patients): hypertension, coronary heart disease (CHD), diabetes mellitus, and prior cerebral infarction; and 3. perioperative data: surgical delay (days from admission to surgery), American Society of Anesthesiologists (ASA) classification, operative duration (minutes), admission complete blood count (CBC) results, postoperative Day 7 CBC results, intraoperative blood loss (mL), and intraoperative blood transfusion volume (mL).

### 2.3 Delirium assessment criteria

Perioperative delirium was diagnosed using the Confusion Assessment Method (CAM) based on the Diagnostic and Statistical Manual of Mental Disorders, Fifth Edition (DSM-5) criteria (17). Assessments were performed twice daily (during morning and evening ward rounds) starting from postoperative day 1 until hospital discharge or postoperative day 7 (whichever occurred first), ensuring comprehensive coverage of the high-risk period for delirium onset. All assessments were conducted independently by a senior attending physician and an experienced nurse. The diagnostic features included the following:

1. Acute onset with fluctuating mental status.
2. Inattention.
3. Disorganized thinking.
4. Altered level of consciousness.

A diagnosis of delirium requires the presence of both features 1 and 2 plus either of features 3 or 4. Patients were stratified into delirium (POD +) and non-delirium (POD-) groups on the basis of perioperative occurrence.

### 2.4 Assessment of perioperative blood loss

Perioperative blood loss was calculated using gross linear equation for erythrocyte volume (18), with the preoperative blood volume (PBV) estimated via the Nadler formula (19). The formulas applied were as follows:

Total blood loss (no transfusion) = PBV × (preoperative HCT − postoperative HCT)/Mean HCT

Total blood loss (with transfusion) = PBV × (preoperative HCT − postoperative HCT)/Mean HCT + transfused volume

### 2.5 Statistical analysis

All analyses were performed with SPSS 24.0 (IBM Corp., United States). Continuous variables are reported as the mean ± standard deviation (x ± s); intergroup comparisons were conducted using independent two-sample *t* tests. Categorical variables are expressed as frequencies (percentages) and were compared via Pearson’s χ^2^ tests. Multicollinearity and model validation: The variance inflation factor (VIF) was computed for all covariates, with a threshold of VIF < 5 indicating acceptable multicollinearity. All the variables satisfied this criterion. Binary logistic regression models were evaluated for goodness-of-fit using the Hosmer–Lemeshow test (*p* = 0.975, > 0.05), confirming appropriate model calibration.

Variable selection strategy: 1. Univariate analysis: Differences between the POD + and POD− groups were compared. 2. Multivariate analysis: Variables with *p* < 0.05 in the univariate analysis were incorporated into a forward stepwise logistic regression to identify independent risk factors for POD. 3. Analysis of Blood Loss and POD Susceptibility: On the basis of the intraoperative blood loss distribution, patients were stratified into four groups using the quartile method. Binary logistic regression was subsequently employed to analyze the association between blood loss levels and postoperative delirium (POD) susceptibility. 4. Visualization: A forest plot was generated using RStudio (v4.3.1) to display adjusted odds ratios (ORs) and 95% confidence intervals (CIs) from the multivariate model.

## 3 Results

### 3.1 Comparison of baseline characteristics

A total of 247 patients were enrolled in this study, including 120 males and 127 females, with a mean age of 80.19 years. Among them, 127 patients had a history of hypertension, 103 had a history of cerebral infarction, 44 had a history of diabetes mellitus, and 97 had a history of coronary heart disease. POD occurred in 98 patients, with an incidence rate of 39.7% (98/247).

No statistically significant differences were detected between the POD + and POD- groups in terms of age (80.42 ± 7.63 vs. 80.04 ± 7.18 years, *p* = 0.693), sex (male/female: 45/53 vs. 75/74, *p* = 0.497), preoperative hemoglobin level (117.52 ± 9.76 vs. 118.76 ± 7.91 g/L, *p* = 0.275), BMI (21.28 ± 2.42 vs. 21.53 ± 2.53, *p* = 0.436), surgical delay (2.41 ± 1.48 vs. 2.52 ± 1.37 days, *p* = 0.556), history of diabetes mellitus (18.37 vs. 17.45%, *p* = 0.854), or history of hypertension (51.02 vs. 51.68%, *p* = 0.919). However, significant differences in the following variables were found between the two groups: history of cerebral infarction (54.08 vs. 33.56%, *p* = 0.001), history of coronary heart disease (53.06 vs. 30.20%, *p* < 0.001), intraoperative blood loss (643.85 ± 64.72 vs. 556.51 ± 73.57 mL, *p* < 0.001), ASA classification > 2 (28.57 vs. 10.07%, *p* < 0.001), and duration of surgery (89.26 ± 17.66 vs. 74.37 ± 10.99 min, *p* < 0.001) (Table 1).

**TABLE 1:** Comparison of baseline characteristics.

Variable	Non-delirium (*n* = 149)	Delirium (*n* = 98)	*P*-value
Blood loss (mL)	556.51 ± 73.57	643.85 ± 64.72	0.00
Age (years)	80.04 ± 7.18	80.42 ± 7.63	0.693
Preoperative hemoglobin (g/L)	118.76 ± 7.92	117.52 ± 9.76	0.275
BMI (kg/m^2^)	21.53 ± 2.53	21.28 ± 2.42	0.436
Operation time (min)	74.37 ± 10.99	89.26 ± 17.66	0.000
Sex (male/female)	75/74	45/53	0.497
Diabetes mellitus	17.45%	18.37%	0.854
Hypertension	51.68%	51.02%	0.919
Cerebral infarction	33.56%	54.08%	0.001
Coronary heart disease	30.20%	53.06%	0.000
ASA > 2	10.07%	28.57%	0.000

### 3.2 Results of the univariate analysis

To analyze the risk factors for POD in elderly patients with hip fractures, univariate logistic regression was performed. The results demonstrated that increased intraoperative blood loss was significantly associated with a greater risk of POD (OR: 6.844, 95% CI: 3.962–11.824; *p* < 0.001). Additionally, prolonged surgical duration (OR: 1.074, 95% CI: 1.051–1.097, *p* = 0.000), history of prior cerebral infarction (OR: 2.332, 95% CI: 1.328–3.934, *p* = 0.001), and history of coronary heart disease (OR: 2.613, 95% CI: 1.540–4.433, *p* < 0.001) were independent risk factors for delirium compared with patients without these comorbidities. Furthermore, ASA classification > 2 was also identified as a significant predictor of POD (Table 2).

**TABLE 2:** Results of the univariate analysis.

Variables	*P*-value	OR	95% CI
Prior cerebral infarction	0.001	2.332	1.328−3.934
ASA > 2	0.000	3.573	1.791−7.128
Coronary heart disease	0.000	2.613	1.540−4.433
Blood loss (per100 mL)	0.000	6.844	3.962−11.824
Surgical duration	0.000	1.074	1.051−1.097

### 3.3 Results of the multivariate logistic regression analysis

**FIGURE 2:**
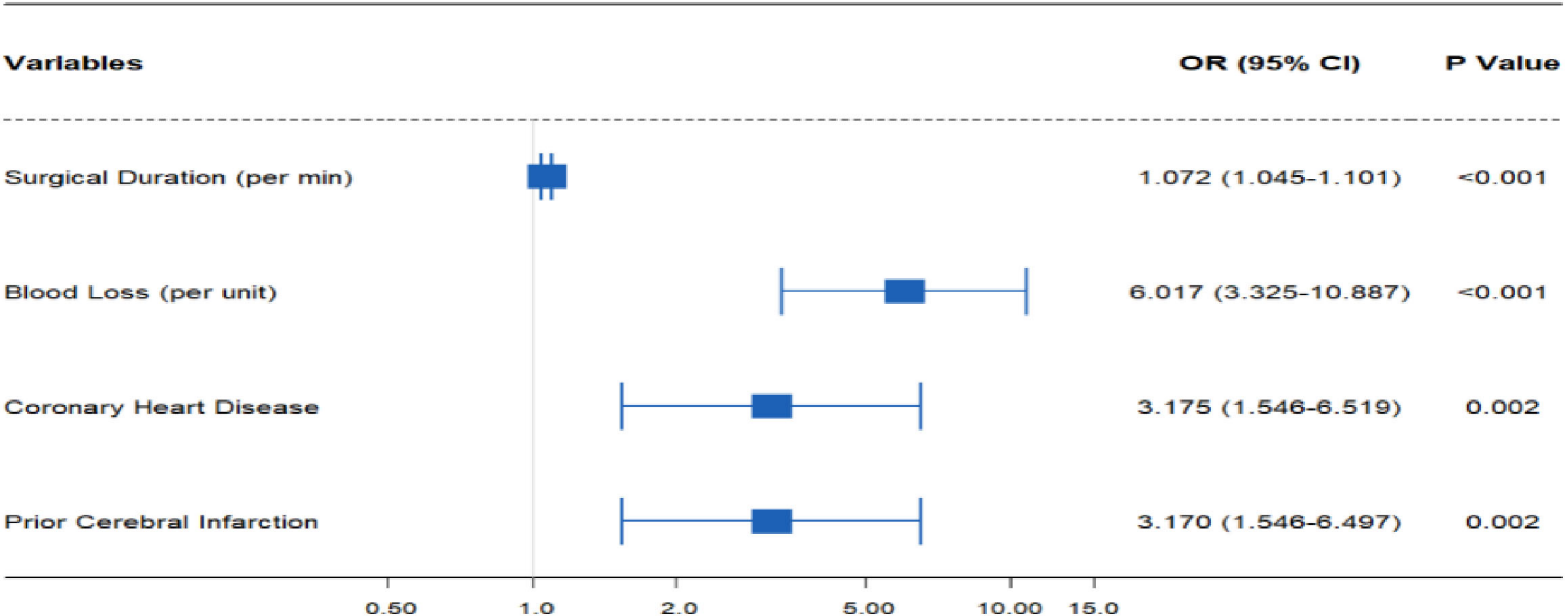
Results of multinomial logistic regression analysis.

A multivariate logistic regression analysis was performed including variables that demonstrated statistical significance in the univariate analysis. The results confirmed that increased intraoperative blood loss (OR: 6.017, 95% CI: 3.325–10.887, *p* < 0.001), prolonged surgical duration (OR: 1.072, 95% CI: 1.045–1.101, *p* < 0.001), history of coronary heart disease (OR: 3.175, 95% CI: 1.546–6.519, *p* = 0.002), and history of prior cerebral infarction (OR: 3.170, 95% CI: 1.546–6.497, *p* = 0.002) were independent risk factors for POD in elderly patients with hip fractures (Table 3 and Figure 2).

**TABLE 3:** Results of the multivariate logistic regression analysis.

Variables	*P*-value	OR	95% CI
Surgical duration	0.000	1.072	1.045−1.101
Blood loss (per 100 mL)	0.000	6.017	3.325−10.887
Coronary heart disease	0.002	3.175	1.546−6.519
Prior cerebral infarction	0.002	3.170	1.546−6.497

### 3.4 Analysis of blood loss and POD susceptibility

**FIGURE 3:**
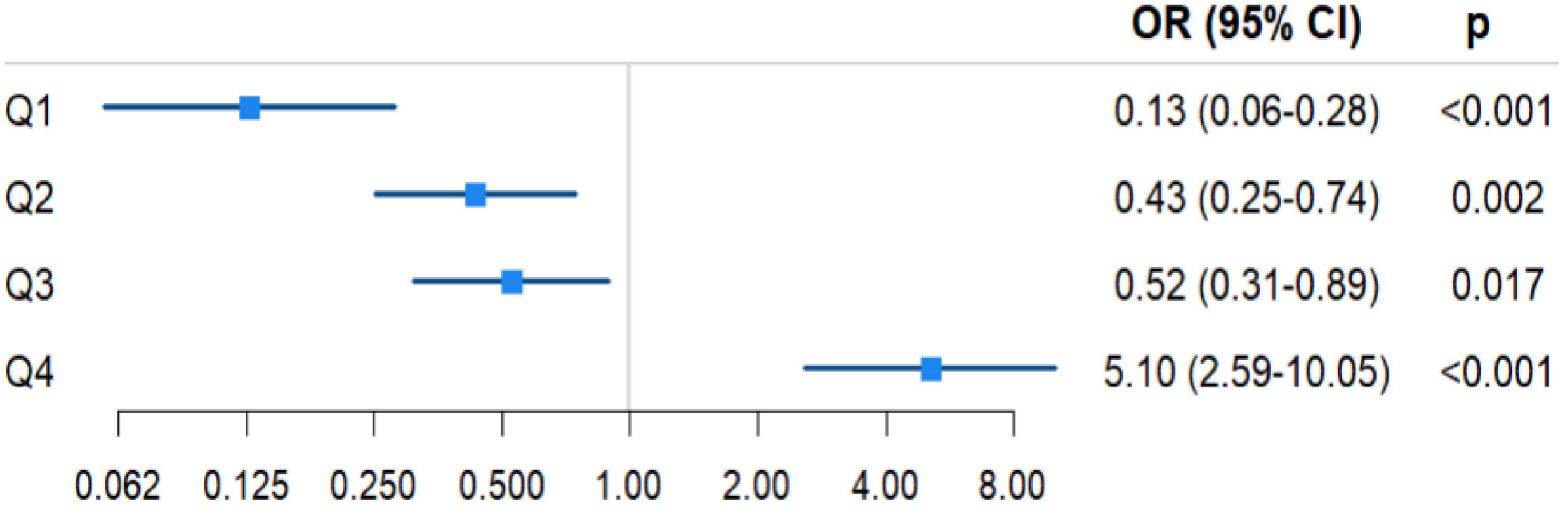
Blood loss stratification and POD association analysis.

Binary logistic regression revealed a significant dose–response relationship between blood loss and POD susceptibility (all *p* < 0.05). Compared with the high blood loss group (Q4, reference), the lower blood loss group presented a progressively lower POD risk: Q1 (OR = 0.127, 95% CI: 0.058–0.279), Q2 (OR = 0.432, 0.252–0.740), and Q3 (OR = 0.525, 0.310–0.891). The high blood loss group had a 5.1-fold greater POD risk (95% CI: 2.589–10.046) (Table 4 and Figure 3).

**TABLE 4:** Association between intraoperative blood loss and POD susceptibility.

Blood loss	β	SE	Wald	*p*	OR	95% CI
Q1 (< 25%)	−2.061	0.401	26.388	< 0.001	0.127	(0.058, 0.279)
Q2 (25%–50%)	−0.840	0.275	9.358	0.002	0.432	(0.252, 0.740)
Q3 (50%–75%)	−0.644	0.269	5.717	0.017	0.525	(0.310, 0.891)
Q4 (> 75%)	1.629	0.346	22.193	< 0.001	5.100	(2.589, 10.046)

## 4 Discussion

The increasing prevalence of hip fractures in the aging population has become a significant public health concern, with surgical intervention serving as the primary therapeutic approach (20, 21). However, postoperative delirium (POD) remains a prevalent and debilitating complication in this population. Geriatric patients undergoing hip fracture surgery are particularly susceptible to POD, with reported incidence rates ranging from 13 to 51% (22, 23). In our cohort of 247 geriatric hip fracture patients, 98 (39.7%) developed POD, which is consistent with existing epidemiological data. Given its association with increased morbidity, prolonged rehabilitation, and elevated health care costs (24), identifying modifiable risk factors is imperative for optimizing patient outcomes (2).

This study demonstrated that perioperative blood loss is an independent risk factor for POD in elderly patients with hip fractures. The underlying pathophysiological mechanisms involve complex multipathway interactions, primarily involving impaired energy metabolism, exacerbation of frailty syndrome, and activation of neuroinflammatory cascades. Perioperative hemorrhage reduces circulating blood volume, resulting in insufficient cerebral oxygen delivery that induces mitochondrial dysfunction and diminished ATP synthesis (5). Subsequent disturbances in neuronal energy metabolism further disrupt the biosynthesis of neurotransmitters, including acetylcholine, thereby directly precipitating delirium. Moreover, the hypoperfusion state secondary to acute blood loss significantly elevates cerebral ischemia risk (2, 3), potentiating POD development. Notably, substantial intraoperative blood loss may aggravate malnutrition in geriatric patients, accelerating the progression of physical frailty. This compromised frailty status reduces physiological reserve capacity, impairing compensatory responses to surgical stress and predisposing patients to cognitive dysfunction (5). Perioperative blood loss can induce neuroinflammation (25), and excessive release of proinflammatory cytokines (e.g., IL-6) may compromise blood–brain barrier (BBB) integrity (4, 26, 27), allowing peripheral inflammatory mediators to infiltrate the central nervous system (CNS). Inflammation-mediated endothelial activation can lead to microthrombus formation (28), further impairing cerebral microcirculation and promoting delirium through dual mechanisms of ischemia and neuronal apoptosis.

This study demonstrated that prolonged surgical duration is an independent risk factor for POD, which aligns with previous findings (25). Extended surgical time may indirectly impair central nervous system function by augmenting systemic stress responses, increasing exposure to anesthetic agents, and promoting the release of inflammatory factors associated with tissue injury. Some studies have employed standardized tools such as the Charlson Comorbidity Index and APACHE II scores to evaluate the association between underlying diseases and postoperative delirium (29), as these metrics hold significant value in assessing baseline health status and predicting delirium risk. However, given the retrospective design of this study and limitations in the completeness of original medical records, we were unable to systematically collect data on these standardized scoring systems. Nevertheless, the study still conducted a comprehensive evaluation based on patients’ comorbidity profiles and ASA > 2 status. A history of old cerebral infarction significantly elevated POD risk (OR = 3.170). Residual neurological damage postinfarction may reduce cerebral tolerance to perioperative hypoxia and metabolic disturbances, whereas chronic cerebral ischemia can exacerbate neuroinflammatory responses and compromise blood–brain barrier integrity, thereby increasing susceptibility to delirium (7). In this study, coronary heart disease (CHD) also emerged as an independent risk factor for POD in elderly hip fracture patients (OR = 3.175, *p* = 0.002), which is consistent with the trend observed in Qi’s research (30). However, direct investigations into the relationship between CHD and POD remain limited. Further studies are warranted to elucidate the underlying mechanisms and clarify potential causal pathways and intervention targets involved. Furthermore, while an ASA classification > 2 was associated with POD occurrence in our study, multivariate analysis revealed that it was not an independent risk factor. This correlation likely reflects the role of the ASA classification as a composite indicator of patients’ baseline health status, incorporating the comorbidity burden and diminished physiological reserve. However, its failure to emerge as an independent predictor in multivariate analysis suggests that the influence of the ASA classification may be superseded by more direct perioperative determinants. This finding aligns with the literature (31), indicating that the ASA classification should be interpreted in conjunction with other specific factors when predicting POD risk. In clinical practice, POD risk assessment should adopt a multidimensional approach rather than relying solely on the ASA classification.

Our findings demonstrate certain discrepancies with those of previous studies regarding age, sex, and BMI as risk factors for POD (13, 32–37). These variations are likely due to our cohort’s demographic homogeneity (mean age: 80.19 years), which attenuated age-related effects. The predominance of frailty and comorbidities (e.g., cerebral infarction, coronary heart disease) may have overshadowed demographic variables, as physiological reserve depletion and comorbid burden likely play a more direct role in delirium pathogenesis than chronological age alon (7, 38). Similarly, BMI and sex were not significantly associated, possibly because modifiable perioperative factors (e.g., blood loss, surgical duration) or balanced intergroup distributions masked their influence. These observations align with emerging evidence that biological age (e.g., frailty, cognitive reserve) may outweigh chronological age in high-risk geriatric populations (39, 40). While age remains a well-established risk factor in broader studies, our results emphasize that in elderly hip fracture patients, perioperative management and comorbid disease optimization may have a greater influence on delirium risk than non-modifiable demographics do. Future research should develop multidimensional risk models that integrate both physiological vulnerability and perioperative stressors, tailored to population-specific characteristics, to better predict and prevent POD in this high-risk group. This study did not include anesthetic techniques as a risk factor for POD due to the predominant use of general anesthesia (GA) in our cohort, with only six patients receiving spinal anesthesia. Current evidence suggests that regional anesthesia (spinal, epidural, or combined techniques without sedation) does not significantly reduce POD incidence compared with GA in elderly hip fracture surgery patients (41, 42).

As a retrospective analysis, this study is inevitably subject to recall bias. Additionally, the relatively small sample size may weaken the associations between study factors and outcomes. In future research, our team plans to conduct prospective cohort studies focusing on perioperative blood loss control strategies, including goal-directed transfusion strategies, intraoperative circulatory monitoring, and the selection of surgical approaches with minimal blood loss for high-risk POD patients. We aimed to evaluate the impact of these interventions on POD incidence and assess their clinical value as modifiable factors. Furthermore, incorporating multicenter collaborations and larger sample sizes may help more accurately validate relevant risk factors and provide more reliable evidence for POD prevention strategies.

In summary, POD in geriatric hip fracture patients has a high incidence rate with complex and multifactorial risk factors. Perioperative blood loss is an independent and modifiable risk factor for postoperative delirium in elderly hip fracture patients. On the basis of these findings, optimizing perioperative management measures—such as reducing intraoperative blood loss and shortening surgical duration—may serve as an effective approach to lowering the incidence of postoperative delirium in this population.

## Data Availability

The original contributions presented in the study are included in the article/supplementary material. Further inquiries can be directed to the corresponding author.
