# Correction: Perioperative blood loss is a risk factor for postoperative delirium in geriatric hip fracture patients: a retrospective study

**DOI:** 10.3389/fmed.2025.1760976

**Published:** 2026-01-05

**Authors:** Frontiers Production Office

**Affiliations:** Frontiers Media SA, Lausanne, Switzerland

**Keywords:** postoperative delirium, hip fracture, geriatrics, blood loss, risk factors

The article type was erroneously given as a Review article. The correct article type is Original Research article.

Accordingly, the below **Ethics statement** was added:

“The studies involving humans were approved by the ethics committee of the Second People's Hospital of Yichang (Approval No. 202523). The studies were conducted in accordance with the local legislation and institutional requirements. Written informed consent to participate in this study was not required from the participants in accordance with the national legislation and the institutional requirements.”

Accordingly, the below **Data availability statement** was added:

“The original contributions presented in the study are included in the article/supplementary material. Further inquiries can be directed to the corresponding author.”

The original version of this article has been updated.

